# Single-dose psilocybin for treatment-resistant obsessive-compulsive disorder: A case report

**DOI:** 10.1016/j.heliyon.2022.e12135

**Published:** 2022-12-06

**Authors:** Benjamin Kelmendi, Stephen A. Kichuk, Giuliana DePalmer, Gayle Maloney, Terence H.W. Ching, Alexander Belser, Christopher Pittenger

**Affiliations:** a Yale School of Medicine, Department of Psychiatry, New Haven, CT, USA; b US Department of Veterans Affairs, National Center for PTSD – Clinical Neuroscience Division, West Haven, CT, USA; c Perth OCD Clinic, Perth, Australia; d Yale University, Department of Psychology, New Haven, CT USA; e Yale Child Study Center, Yale University School of Medicine, New Haven, CT, USA; f Yale Center for Brain and Mind Health, Yale University School of Medicine, New Haven, CT, USA

**Keywords:** Obsessive-compulsive disorder (OCD), Psilocybin, Case report, Treatment

## Abstract

Classic psychedelics, such as psilocybin, act on the brain's serotonin system and produce striking psychological effects. Early work in the 1950s and 1960s and more recent controlled studies suggest benefit from psychedelic treatment in a number of conditions. A few case reports in recreational users and a single experimental study suggest benefit in patients with obsessive-compulsive disorder (OCD), but careful clinical data and long-term follow-up have been lacking. Here we describe a case of a patient with refractory OCD treated with psilocybin and followed prospectively for a year, with marked symptomatic improvement. We provide qualitative and quantitative detail of his experience during and after treatment. Improvement in OCD symptoms (YBOCS declined from 24 to 0–2) was accompanied by broader changes in his relationship to his emotions, social and work function, and quality of life. This individual was an early participant in an ongoing controlled study of psilocybin in the treatment of OCD (NCT03356483). These results are preliminary but promising, motivating ongoing investigations of the therapeutic potential of appropriately monitored and supported psychedelic treatment in the treatment of patients with obsessions and compulsions.

## 1 Introduction

### 1.1 Research and theoretical basis for psilocybin treatment

Although empirically supported treatments for obsessive-compulsive disorder (OCD) greatly benefit many patients, a significant proportion experience persisting symptoms and impairment even after intensive treatments (Pittenger, 2021). Thus, there is an urgent need to explore innovative therapeutic approaches for treatment-resistant OCD.

There has been renewed interest in recent years in the therapeutic potential of classic psychedelic substances, especially psilocybin (the primary active component in *Psilocybe* mushrooms), for various psychiatric disorders. Recent controlled studies suggest benefit after one or two doses of psilocybin in major depressive disorder (Davis et al., 2021; Carhart-Harris et al., 2016, 2021) and in depressive and anxiety symptoms in patients with late-stage cancer (Grob et al., 2011; Griffiths et al., 2016; Ross et al., 2016). Larger multi-site studies are underway.

Research on the use of psilocybin in the treatment of OCD is more limited. Early case reports suggested improvement in OCD symptoms after use of psilocybin and LSD (Leonard and Rappoport, 1987; Moreno and Delgado, 1997). Later, Moreno et al. (2006) reported findings from a multi-dose, partially blinded crossover study, in which nine adults with OCD showed marked improvement in symptom severity (23–100% decreases in Yale-Brown Obsessive-Compulsive Scale [Y-BOCS; Goodman et al., 1989a; Goodman et al., 1989b] scores) at 24-hour follow-up, with no difference between doses. More recently, two case reports indicating benefit of psilocybin for OCD have been published (Lugo-Radillo and Cortes-Lopez, 2021; Wilcox, 2014). Meanwhile, anecdotes from individuals who have self-medicated with psilocybin to address their OCD symptoms continue to appear in various discussion forums (e.g., www.erowid.org). Careful study is needed to follow up on these observations.

We have begun a double-blinded, randomized placebo-controlled trial of single-dose psilocybin in treatment-resistant OCD (NCT03356483). Here, we report the experiences of an early participant in this trial who experienced rapid and lasting improvement. It is important to note that this response is not typical – we have seen cases in which improvement was less dramatic, and cases in which there was no significant change at all. Our goal in presenting detailed qualitative data here is to provide the field with insight into the subjective experience and correlates of change in a responder, for the benefit of clinicians interested in such work and to inform theories about underlying psychological and neural mechanisms.

Various theoretical rationales for the use of psilocybin in the treatment of OCD have been proposed, at different levels of analysis. At the neurotransmitter level, serotonin reuptake inhibitors are the best-supported class of pharmacotherapy for OCD (Pittenger and Bloch, 2014). Since psilocybin and other classic psychedelics act on the serotonin system (Halberstadt, 2015), some suggest that they may activate overlapping cellular processes (Pittenger, 2021). As a variation on this theme, the primary action of psychedelic substances on serotonin receptors may lead to a secondary glutamate surge (Pittenger, 2021; Mason et al., 2020). Glutamate dysregulation has received much attention as a potential pathophysiological contributor and treatment target in OCD (Pittenger, 2015; Pittenger et al., 2011), and so these glutamatergic effects might produce therapeutic benefit. Downstream effects of neurotransmitter modulation may include growth of new synapses (Shao et al., 2021) or genomic effects such as reversal of aging-associated processes (Germann et al., 2020).

At the brain network level, psychedelics may acutely and adaptively modulate the default mode network, in which dysregulation has been proposed to contribute to many forms of psychopathology (Carhart-Harris et al., 2017; Smigielski et al., 2019). Changes in this circuitry are associated with treatment response in OCD (Reggente et al., 2018), and so reorganization of this network and its relationship with other major brain networks (Adams et al., 2022) after psychedelic treatment may have therapeutic benefit (Romeo et al., 2021).

At the psychological level, the acute mystical experiences (e.g., of ego dissolution) evoked by psychedelics have been shown to correlate with symptom improvement in several clinical contexts (Romeo et al., 2021). It has been suggested that reintegration following this dissolution may lead to enhanced cognitive flexibility (Carhart-Harris and Nutt, 2017), which could be therapeutic in OCD. Indeed, Doss et al. (2021) found that psilocybin therapy increased cognitive flexibility, as measured in terms of perseverative errors on a set-shifting task, for at least 4 weeks post-treatment in major depressive disorder. It has also been suggested that psychedelics can enhance creativity (Germann, 2019); this, to, might contribute to therapeutic change.

At a more basic psychological level, the potent thoughts, feelings, and memories that emerge during a psychedelic experience may provide an opportunity to form both new insights and new ways of relating to and responding to inner experience. For instance, patients may learn a process of awareness, acceptance, approach, and “letting go” of emotions to replace a rigid emphasis on controlling, suppressing, or avoiding them (Watts et al., 2017). Emotion regulation difficulties are associated with OCD (Berman et al., 2018; Khosravani et al., 2020a; Khosravani et al., 2020b; Stern et al., 2014; Yap et al., 2018), so an important part of treatment may be to help people with OCD better process and respond to their emotions.

## 2 Case profile

Daniel (a pseudonym) was a 33-year-old man who presented for participation in an ongoing randomized controlled trial of psilocybin for treatment-resistant OCD (NCT03356483).

Daniel reported a primary diagnosis of OCD and a history of a major depressive episode, Tourette syndrome (TS), and panic disorder. The diagnosis of OCD was confirmed by a doctoral-level clinician (BK) and an experienced interviewer (SK) using the Mini International Neuropsychiatric Interview, Version 7 (MINI-7; Sheehan et al., 1998). Daniel was first diagnosed with OCD and TS at about age 10. Despite multiple trials of selective serotonin reuptake inhibitors and cognitive-behavioral therapy, Daniel reported never achieving adequate relief of OCD symptoms. His severity score on the Y-BOCS at the time of initial evaluation was 24, which falls within the 'severe' range.

Although Daniel reported some success in coping with his OCD symptoms using compensatory strategies and personal resources (e.g., general intelligence, creativeness, and persistence at tasks), he continued to struggle in managing his emotions. He found his social functioning, hobbies, and work to be detrimentally affected because of both his OCD symptoms and his difficulties managing emotions.

Daniel reported OCD symptoms since his early teens, with multiple episodes of treatment and limited response to both psychotherapy and pharmacotherapy. He reported four past exposures to psilocybin mushrooms, twice in his early 20s and twice about 2.5 years prior to his participation in the current study. He reported finding that these mushrooms provided some help for OCD symptoms in that they reduced “static” in his mind. These benefits were transient, lasting 1–2 weeks, and did not occur every time. He reported that effects appeared related to his mental preparation. He stated, “I recall one instance where I expected some relief and received none. I hadn't put anything into the process and so the drug didn't give anything back.”

He also reported a single use of LSD, about 1.5 years prior to participation in the current study; he reported minimal, if any, benefit to OCD symptoms from this experience. At the outset of the current study he was hopeful that treatment with psilocybin would provide some symptom improvement but expressed a realistic understanding that it was unproven and the benefits, if any, were impossible to predict.

### 2.1 Study protocol

This study has been approved by the Yale Human Investigations Committee (HIC #2000020355) and is conducted under an Investigational New Drug (IND) permit from the U.S. Food and Drug Administration (IND # 34406), under the Schedule 1 DEA certificate held by corresponding author (BK). The participant consented to the description of his treatment in this report.

Daniel worked with two study facilitators (SK and GD), both of whom had Master's degrees, extensive experience working with individuals with psychiatric symptoms (especially OCD), workshop training in the facilitation of psilocybin dosing sessions, and prior experience facilitating dosing sessions under the supervision of an experienced doctoral-level facilitator.

Daniel was randomized to receive psilocybin; dosage was 0.25 mg/kg (19.4mg psilocybin). Psilocybin (Kargbo et al., 2020) was provided by the Zeeh Pharmaceutical Experiment Station at the University of Wisconsin (https://pharmacy.wisc.edu/centers/zeeh-pharmaceutical-experiment-station). This dose is in the middle of the range (0.20–0.30 mg/kg) that typically produces a robust psychedelic effect and has been used in other recent therapeutic studies and falls between the higher two doses used in the one previous study of psilocybin in OCD (Moreno et al., 2006).

Daniel, the facilitators and the study rater were blinded to condition (psilocybin vs niacin 250 mg) at the time of dose administration. The primary outcome measure for this first controlled study of psilocybin in the treatment of OCD is 48 h after dosing; this time point was chosen to focus on rapid symptom changes and so as to have our primary outcome uncontaminated by retrospective psychological processing during integration sessions, which began after 48 h. Daniel and the facilitators were formally unblinded to dosing condition at 48 h post-dosing. The study rater was unblinded at one-week follow-up, because participants who received placebo were not followed beyond this point (they were offered the opportunity to receive open-label psilocybin). The facilitators conducted follow-up psychological integration sessions at 48 h and at one, two, and 12 weeks post-dosing. Clinical assessments and ratings were conducted at 48 h post-dose (primary outcome), then at one week, and at two, four, eight, and 12 weeks. All sessions were recorded and later transcribed, permitting the extensive verbatim quotes provided in this report.

The facilitators spent time getting to know Daniel prior to the dosing session and provided nondirective support during the session, in line with past work that has suggested the maintenance of an environment of psychological safety during dosing can help to prevent ‘bad trips’ (Johnson et al., 2008). No structured psychotherapy was provided before, during, or after dosing, as we do not assume that mechanisms of change following psilocybin dosing align with those underlying the efficacy of evidence-based therapies for OCD. Integrating psychedelic dosing with structured psychotherapy in OCD is an important topic for future research.

### 2.2 Course of treatment

#### 2.2.1 Preparatory sessions

In preparatory sessions, the facilitators provided guidance for the upcoming dosing session. This included discussion of session procedures, psychoeducation on the range of possible acute effects of psilocybin, and the potential for clinical change with either psilocybin or placebo. Responsibilities of facilitators were discussed, and there was a focus on developing trust and rapport between the study participant and the facilitators. Additionally, the facilitators provided guidance on how to approach any experiences that might arise during dosing. The aim was to foster within Daniel a curiosity about the contents of his mind and a willingness to accept and “walk into” whatever thoughts or feelings might emerge during the dosing session.

The facilitators also explored Daniel's behavioral and cognitive patterns, his relationship with his emotions, and themes important to his overall functioning. There was some exploration of his OCD symptoms, the concerns and motivations underlying them, and the impact of these symptoms on his life. However, only a minority of preparation time was focused specifically on OCD symptoms, and they were not directly targeted for change. In the facilitators' view, Daniel's general patterns of relating to and processing his emotions were an especially important target. The predominant focus in preparatory sessions was thus on teaching Daniel about the nature, purpose, and constructive use of emotions.

As the facilitators explored Daniel's history, he showed great discomfort with the experience of negative emotions and a lifelong pattern of “compacting” and avoiding his emotions. This repeatedly led to these emotions building up and overwhelming him, and he described himself as emotionally “fragile.” He also described extensively analyzing situations to identify what he “should” be feeling or doing, then trying to steer his feelings and actions based upon these formulations. He was troubled by questions of how many of his life choices and circumstances were dictated by these formulations rather than by his authentic desires. Further, he perceived a troubling incongruity between what he tended to think he should feel versus the feelings he actually had. He also had substantial difficulty sharing feelings with others and tended not to disclose them. As a result, he lacked the kinds of deeper connection with others he desired and believed he was not truly understood as a person.

The facilitators explored meaningful episodes in his life to uncover what was important and personally fulfilling for him. They paired this with an exploration of how he wanted to live his life moving forward. Daniel noted that, during the most meaningful times in his life, his “guards were down,” he was “okay with feeling,” and felt “almost OCD-free.” The facilitators highlighted how he was neither suppressing nor avoiding his emotions during those times, but was, instead, open to and connected with them. The facilitators also highlighted his intelligence, curiosity, and creativity and explored how he might apply these qualities to his life alongside an openness to his emotions.

Near the close of the second preparatory session, an intention for the dosing session was established to anchor his experience. By this point, Daniel expressed a desire to “slow down and let myself feel things” and “to be more honest about what moves me.” Proceeding from this desire, he developed an intention for the dosing session to be open to the breadth and depth of his feelings, and to be honest with both himself and others about them.

#### 2.2.2 Psilocybin dosing

The dosing session was conducted within a research-dedicated psychiatric inpatient unit. The treatment room itself was modified to be conducive to relaxation. Distracting external environmental stimuli were minimized. During dosing, Daniel laid down on a pull-out couch, with the facilitators seated nearby. Through most of the experience, he wore eyeshades and headphones with music, though he was free to remove them and interact with facilitators at will. The facilitators provided support and guidance when needed.

At the start of the dosing session, the facilitators assured Daniel that “anything and everything is welcome” and reminded him of his intention to let himself openly feel and share his emotions. Discussion ensued about his thoughts following the preparatory sessions. He shared an insight that he had been trying to “intellectualize” for a long time and had not fully grasped prior to this time, that “being vulnerable is a recoverable state” (e.g., that he can recover from challenging or vulnerable feelings).

Approximately 90 min after ingestion of psilocybin, the study drug began exerting clear psychological effects. Daniel first reported feeling “spacey” and then entering an “interesting headspace.” He was encouraged to put eyeshades and headphones on and allow the experience to unfold. As his thoughts and emotions began to pick up force, Daniel initially felt resistant to this process due to his strongly learned tendency to push away his feelings. However, at this moment, he recalled being encouraged in preparatory sessions to openly experience whatever might arise during his dosing session. He viewed this prior (and ongoing) guidance to be important in enabling the psychedelic process to unfold.

Over a period of about 7 h, Daniel experienced a range of deep and intense emotions. He reported moving into an experience of death where he “went to a place beyond time” in which there was “neither calm nor the absence of calm.” In this experience, he reported feeling his “whole body compost into the ground.” He then reported experiencing a rebirth in which he went “from nothingness into something,” and described “growing back into myself.” This process first involved an experience of growing into a sapling, then a mature tree, living through multiple seasons, and having the ability to be fully present at each moment. After this, he experienced being reborn as a human being. He relived memories, was present with and appreciated each moment and accompanying feelings (both positive and negative), and realized that trying to avoid or suppress feelings could inadvertently lead to greater problems later on. He realized that he had been living as if “chasing perfection was the answer” to “being happy” or “living a life.”

At one point during the dosing session, Daniel stated aloud, “This is giving me my life back.” Later on, as the psilocybin's effects wore diminishing, Daniel described the psychedelic experience he'd just been through as the most intense cascade of emotions he had ever felt. Having come through it, he expressed that, however memories or emotions may feel in their quality or intensity, he can experience them and still be okay. He also expressed that he now felt fully opened to his feelings, wanted to share them with others, and was confident in his ability to do so. As he said, “I can carry forward in a way that's acceptable to me. And I think that will be acceptable to others.” He further noted that he had more work and learning ahead and was committed to the process.

#### 2.2.3 Integration sessions

Four integration sessions were conducted by the study facilitators: 48 h, one week, two weeks, and twelve weeks after dosing. Integration sessions were aimed at reinforcing guidance provided in preparatory sessions, consolidating lessons learned in the dosing session, extracting new insights, and incorporating these lessons into daily life. As in preparation, psychoeducation about emotions was provided throughout.

Starting with the first integration at 48 h and onward, Daniel reported a new “space” separating him from his OCD symptoms and a changed relationship with them. They now felt “vestigial”, and he was readily able to shift from obsessive thoughts onto other thoughts and behaviors. He described each day as providing new opportunities to dismiss any remaining symptoms and that “making those corrections is not a big deal” with the “distance and perspective” he now had.

Daniel described that, prior to the study, a lack of emotional processing of everyday occurrences would lead to “the fallout” that was obsessive thinking. Essentially, instead of processing an event, he described becoming fixated on specific components of events, and then obsessing about those components. However, when he now experiences discomfort from daily events that would have previously triggered that “fallout,” he described instead engaging in a conscious, effortful process of recognizing his feelings, processing them, and moving on. “That's never happened before,” he stated. He also said of the basic experience of emotion, “To use that as constructive input is totally new for me.”

He reported long being afraid of whether he and his basic experience of life were “acceptable” and feeling that maybe he was only “safe” because of his long-standing behavioral patterns. During his psychedelic experience, however, he experienced what he described as a collapse down to the very “nucleus” of himself. From this, along with his experience of “being a tree,” he came to appreciate that existence itself is inherently meaningful, and therefore acceptable and worthy of embrace. He also described it as “the linchpin” of enabling him to shift away from his old patterns. Daniel explained, “Having this perspective of having been a different thing completely, and of having been nothing [in that space beyond time] is what… allows me to let go and to say, ‘Okay, that's why this stuff feels vestigial. That's why it feels like a habit in a body and in a brain… and [why I can now] break some of those habits.”

He experienced the preparatory sessions and intention-setting for the dosing session as important in providing a mental framework for his psychedelic experience. He also found it critically important that the preparatory sessions had not been narrowly focused on his OCD symptoms but rather on their broader psychological and emotional context. This, along with his perceived safety and comfort with his facilitators, enabled him to be emotionally open and “go to a completely different place.” He expressed a belief that without such careful preparation, he wouldn't have had the same experience and instead might have had a “bad trip.”

The various aspects of his psychedelic experience “informed one another,” he felt, and showed to him that his stated intention to allow himself to feel openly and fully was something he could now *actually* do. This was a novel experience for him. The intensity of his emotions, along with his openness to them, showed him that, as he stated, “I didn't have to be afraid of my experiences and the feelings they evoked. I didn't have to keep running.” He recognized that his history of “compacting” and avoiding feelings prevented him from processing them. Since the dosing session, he was no longer so intently focused on what was “wrong” in his external surroundings. Instead, he now described himself as “re-contextualizing” his feelings and being more aware of and present with them as they occurred. He was accepting of them and expressed an intent to “listen better” to what they might bring. Additionally, Daniel revised his previous feeling of being a “fragile person”, and he now saw himself instead as a “receptive person.” He stated, “I'm not literally a new person, but more in touch with who I am.”

#### 2.2.4 One-month post-dosing qualitative interview

In a qualitative interview with an independent evaluator one month after dosing, Daniel reiterated the major insights detailed above regarding his experiences in the preparatory, dosing, and integration sessions. His reporting demonstrated the consistency and durability of these insights. Importantly, he expressed the insight that his OCD arose in part out of non-acceptance of fears in life, which then provided an opportunity for the OCD to “take hold.” He also reported the insight that it was his previous relationship with his thoughts (i.e., obsessing, worrying) which was problematic, instead of the contents of his thoughts per se. He described progressively practicing non-judgmental mindful acceptance of his thoughts and emotions as they arose. He also described becoming increasingly able to simply acknowledge that they exist instead of delving deeper into them. He reported that through this approach, he was able to prevent the onset of most, if not all, of his obsessive cascades, thus “reclaiming” more time in his day. He expressed the belief that his experience of “dying” in his psilocybin session was essential for him to develop the ability to accept his obsessive fears. This, he felt, was because he had already confronted one of the deepest human fears, that of death. He also viewed “being a tree” as another “pivotal” moment, one that inspired him to practice “letting go” and be non-judgmental of his negative internal experiences.

Overall, Daniel's reported increased cognitive and emotional flexibility seemed to pervade his everyday life one-month post-dosing. He described a more flexible approach to living, in which he attuned to his body more and adjusted accordingly (e.g., eating meat again after a year of being a vegetarian). This was a notable change from before, where he would rigidly follow a set routine as demanded by his OCD symptoms. At the end of the interview, Daniel described associating little to no stigma with having (or more precisely, having had) OCD; he was actively discussing it with his friends, acquaintances, and loved ones, instead of concealing it. He reported hoping that others with OCD will be able to similarly accept that they need help and seek treatment, whether it involves psilocybin therapy or not.

## 3 Outcomes

At 12 weeks post-dose, Daniel reported that OCD was no longer a significant part of his life, despite having a persistent “imprint” of obsessions and compulsions in his mind. He felt that if he didn't continue to work at managing this imprint, he could “slip back,” but even if that happened, he now had the “tools” to manage it. Referring to his OCD, he said, “Even if this were to come back, I'm on it.”

**Figure 1:**
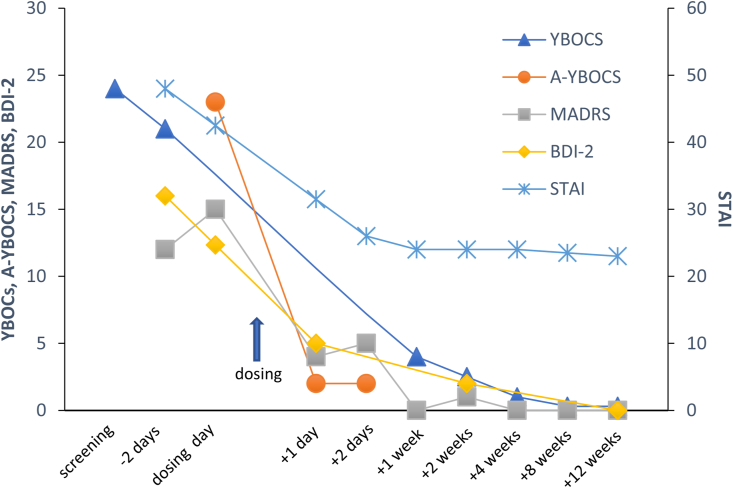
Symptom change over time. YBOCS = Yale-Brown Obsessive-Compulsive Scale; A-YBOCS = Acute Yale-Brown Obsessive-Compulsive Scale; MADRS = Montgomery-Asberg Depression Rating Scale; STAI = State-Trait Anxiety Inventory; BDI-II = Beck Depression Inventory – Second Edition.

**Figure 2:**
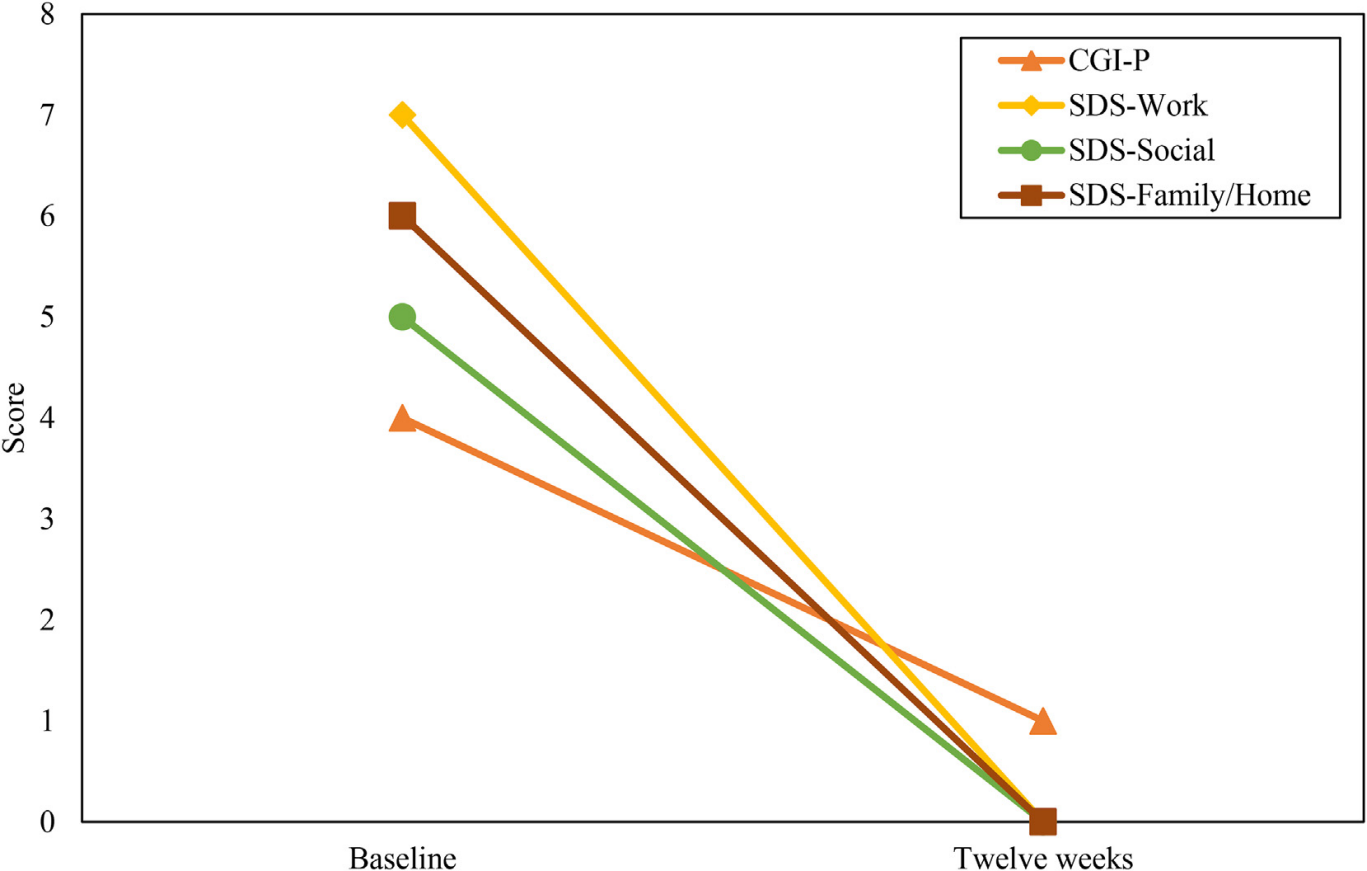
Functioning-related outcomes across time for RL. CGI-S = Clinical Global Impression - Severity; SDS = Sheehan Disability Scale.

Daniel's Y-BOCS severity score declined slightly, from 24 to 21, between initial evaluation and baseline, two days prior to dosing; this may reflect increased comfort with the environment and with clinic staff, positive expectations of the dosing session, or simply a shift in focus from his usual preoccupations to the upcoming treatment session. There was a decline in the acute YBOCS (A-YBOCS, which is similar to the YBOCS but asks subjects to describe symptoms over the previous 24 h rather than the previous week; Bloch et al., 2012) from 23 on the morning of the dosing session to 2 at 48 h post-dosing (primary outcome measure, collected prior to the first integration session). YBOCS was 4 at one week, 1 at four weeks, 1 at eight weeks, and 0 at 12 weeks. Symptoms in other measured domains also showed improvements, including depressive symptoms (on the Beck Depression Inventory – Second Edition [BDI-II; Beck et al., 1996] and Montgomery-Asberg Depression Rating Scale [MADRS; Montgomery and Asberg, 1979]), anxiety symptoms (on the State-Trait Anxiety Inventory [STAI]; Spielberger et al., 1983), global symptom severity (on the Clinical Global Impressions Scale [CGI]), and overall disability (Sheehan Disability Scale [SDS]; Sheehan, 2000). It is notable that these improvements persisted up to 12 weeks post-dosing, despite the fact that psilocybin was administered only once. These scores are shown in Figures 1 and 2.

Daniel described a sustained improvement in his ability to be present with his feelings and use them constructively, and a sense that forgiveness had replaced his previous self-judgment. He described an ability to embrace himself, his inner experience, and his surroundings in ways that he had previously struggled with. A sense of curiosity about his surroundings replaced his previous sense that something was somehow wrong.

Psychosocially, his relationship with his supportive wife had improved substantially. He described a history of chronically relying on her for emotional support and difficulty with offering the same in return. However, he now reported being able to give her support in a way he never had before and that this added a new dimension to their relationship. He additionally felt he was in a place where he could be a better father. He found himself more able to express his feelings and communicate with other family members as well. Beyond his familial relationships, he noted that friends were perceiving positive change in him and were even asking for advice about wellness and change, which was a novel experience. At 12 weeks post-dosing, he took on a new job from which he had previously held back on due to fears of the stress it would entail. He stated that, in his view, he wasn't just doing better than the average person with OCD, he was “doing better than your average Joe.” He further affirmed, “This was a very powerful inflection point in my life.”

### 3.1 One-year post-dosing follow-up

Although Daniel's study participation concluded at 12-weeks post-dosing (the full study duration), study facilitators were in contact with him regarding this report at one-year post-dosing. Daniel reported having observed, with his wife, the anniversary of his psychedelic experience as his “re-birthday.” He also stated that, “…My world continues to look and feel radically different than it did a year ago. I'm still OCD-free and have a completely new relationship with my emotions.”

Between 9 months and 1.5 years post-dosing, Daniel experienced two significantly stressful life events. The first of these events elicited some strong and challenging emotions, but he reported active efforts to acknowledge and accept them rather than avoid them. He was able to recover without great difficulty.

The second event was substantially more stressful for him. The aftermath of this event brought about, as he described, a “crushing anxiety” and a recurrence of significant OCD symptoms. He felt “scared” that this might represent “the beginning of a slide” into his pre-study state.

He reported that after several days of this, he recognized what was happening and “hit the brakes hard.” For about four days, he dedicated significant time to processing the emotions he felt were stemming from the stressful event. He reported applying lessons learned from both the preparation/integration work with his facilitators and from the psychedelic experience itself. He described allowing his emotions to be present, along with accepting and feeling them without trying to suppress or avoid them. He emphasized that it was “not a cognitive process”. He continued these efforts over a period of about two weeks. As he processed his emotions, he stated, “the anxiety and then the OCD faded.”

Daniel remarked that effectively addressing this symptom recurrence highlighted for him that he “still carried the toolkit” of insights and skills he learned from his experience in the psilocybin treatment study. He feels he can apply it at “any time” he needs. He described that the change he experienced through the study “wasn't only a singular transformation” but that the “toolkit” also “provided a sustainable means for ongoing transformation.”

In working with Daniel, the facilitators observed his strongly engaged and active efforts from the beginning of preparatory sessions. He reported maintaining consistent efforts to nurture his mental health well-beyond the end of his study participation. Daniel has emphasized that such continual, active effort “is required to sustain” his mental health. He noted also how the facilitators had framed change and good mental health as a long-term process, and that this framing was important to him. As he stated, they “didn't treat it like a light switch, so I didn't treat it like a light switch.”

## 4 Discussion

We have described a case of marked and sustained improvement in a person with longstanding treatment-resistant OCD after a single dose of psilocybin, paired with general psychological guidance and support, and delivered in a controlled setting. This report is consistent with recent findings of sustained improvement after one or two doses of psilocybin in major depressive disorder (Carhart-Harris et al., 2016; Carhart-Harris et al., 2021; Davis et al., 2021) and in depression and anxiety symptoms in individuals with late-stage cancer (Grob et al., 2011; Griffiths et al., 2016; Ross et al., 2016).

Our findings are also consistent with previous case reports and a single open-label trial among individuals with OCD. However, previous case reports have retrospectively described clinical benefit in individuals using illegally acquired Psilocybe mushrooms (Leonard and Rapoport, 1987; Lugo-Radillo and Cortes-Lopez, 2021; Moreno and Delgado, 1997; Wilcox, 2014). Dose and purity are difficult to assess in such cases, and most of the individuals described were also using complex mixtures of other psychoactive substances of indeterminable dosages. In contrast, we used highly purified psilocybin in a carefully screened individual taking no other medications and using no other psychoactive or illicit substances. A previously described case series indicated clinical benefit from several different doses of psilocybin administered prospectively using a crossover design, but follow-up was limited to 24 h. In contrast, we followed this person prospectively for 12 weeks, and intermittently for 18 months, and observed sustained improvement. In addition to reporting objective measures of symptom change, we have provided a detailed qualitative description and include excerpts from this person's report of his experience and his understanding of the changes he experienced. It is our hope that these details will be of use to others considering similar research, or theorizing about the mechanisms of therapeutic change in psilocybin-based treatments.

Importantly, psilocybin administration was coupled with a considerable amount of psychological guidance and support – before, during, and after psilocybin administration. While this intervention was not specific to OCD symptomatology, its potential impact on OCD symptoms should not be underestimated. Its importance was explicitly noted, retrospectively, by the patient. An individual's preparation, aims, attitudes, and physical and sociocultural environment (referred to as “set and setting”) may interact with psychedelic effects in influential ways throughout the psychedelic experience (Carhart-Harris et al., 2018; Hartogsohn, 2016). The importance of such variables in the development of a therapeutic response to psilocybin treatment remains to be thoroughly elucidated. Our strategy has been to provide broad psychological guidance and support to people with OCD receiving psilocybin – not as directive as would be present in a structured psychotherapy program, but more than a simple pharmacological administration. Exploring the interaction between pharmacological and contextual variables in psychedelic treatment is a complex but critical direction for future research.

## 5 Implications and recommendations

The past decade has seen renewed interest in the potential of psychedelic medicines to treat psychiatric symptoms and enhance psychological well-being. Our observations in the current case support the possibility that psilocybin-based treatment may be of benefit to some individuals with treatment-resistant OCD.

That said, it would be premature to suggest broad use of this treatment strategy, for several reasons. First, while there is optimism that the regulatory environment will change in the coming years, psilocybin and other psychedelics remain Schedule 1 substances in the United States, and are similarly restricted in most other jurisdictions. Their use is complicated by this regulatory landscape and is currently illegal in most contexts outside of the research setting. It is unclear if, how, or when the clinical use of psychedelic compounds outside of restricted research settings will become available. Second, the enormous optimism currently attending the use of psychedelic substances should not obscure how little we know about their optimal deployment, presuming that the field ultimately reaches that point. Questions of dosing (both dosage and frequency) and of the optimal therapeutic accompaniments, as discussed above, remain to be definitively answered and may differ among diagnoses.

Finally, the optimal target population for psychedelic treatment is unclear. Importantly, controlled studies such as ours are subject to a high level of regulation and scrutiny, and participants enrolled into such studies may not be representative of the broader population with a particular diagnosis. It has become conventional practice in psilocybin trials to exclude individuals with a history or risk for psychosis, active substance use, polypharmacy, active suicidality, and diagnostic complexity. If and when the regulatory environment relaxes and psilocybin becomes available and appropriate for use outside of heavily regulated research settings, it will be critical to carefully map out the appropriate boundaries of the target clinical population and to be alert and responsive to idiosyncratic needs of particular patient groups (Neitzke-Spruill, 2020).

In sum, while our observations in this case are promising, careful further work is needed. Daniel was an early participant in our ongoing double-blinded, randomized placebo-controlled study of psilocybin in individuals with treatment-resistant OCD (NCT03356483). Two other studies of psilocybin treatment in OCD are in their early phases (NCT03300947; NCT04882839), and others are being planned. We hope that, in the coming years, the role of psilocybin and other psychedelic treatments in patients with OCD will be clarified. We also hope that new treatment options become available for the significant fraction of people with OCD whose symptoms remain refractory to standard treatments.

## Declarations

### Author contribution statement

All authors listed have significantly contributed to the investigation, development and writing of this article.

### Funding statement

This work was supported by a grant from Yale Center for Clinical Investigation, grants from the Heffter Foundation and the Turnbull Family Foundation, and by the State of Connecticut through its support of the Ribicoff Research Facilities at the Connecticut Mental Health Center.

### Data availability statement

Data included in article/supp. material/referenced in article.

### Declaration of interests statement

The authors declare the following conflict of interests:

Benjamin Kelmendi is co-founder and Chief Scientific Advisor for Transcend Therapeutics and has consulted for Ceruvia Lifesciences and Lobe Sciences.

Christopher Pittenger serves as a consultant for Biohaven, Teva, Lundbeck, Brainsway, Ceruvia Lifesciences, Transcend Pharmaceuticals, Nobilis Therapeutics, and Freedom Biotech, receives royalties and/or honoraria from Oxford University Press and Elsevier, and has filed a patent on the use of neurofeedback in the treatment of anxiety, which is not relevant to the current work.

Alexander Belser became Cybin's Chief Clinical Officer during the course of the present study, and subsequently had no further role in the conduct or analysis of the study or in manuscript development. In the last 3 years, A.B. has also received financial compensation for psychedelic research-related activities from MAPS Public Benefit Corporation, Synthesis Institute, Chacruna Institute, and Adelia Therapeutics and has filed patents on the use of psychedelic compounds for the treatment of psychiatric indications.

Christopher Pittenger and Benjamin Kelmendi have filed a patent on the use of psilocybin in the treatment of obsessive-compulsive disorder (#US17/466,111).

### Additional information

No additional information is available for this paper.
